# Seroconversion and seroprevalence of TORCH infections in a pregnant women cohort study, Mombasa, Kenya, 2017–2019

**DOI:** 10.1017/S0950268824000165

**Published:** 2024-02-02

**Authors:** Elizabeth Hunsperger, Eric Osoro, Peninah Munyua, M. Kariuki Njenga, Harriet Mirieri, Gilbert Kikwai, Dennis Odhiambo, Moshe Dayan, Victor Omballa, George O. Agogo, Cyrus Mugo, Marc-Alain Widdowson, Irene Inwani

**Affiliations:** 1 Division of Global Health Protection, US Centers for Disease Control and Prevention (CDC), Nairobi, Kenya; 2 Washington State University (WSU) Global Health Kenya, Nairobi, Kenya; 3 Paul G. Allen School for Global Health, Washington State University (WSU), Pullman, WA, USA; 4 Kenya Medical Research Institute (KEMRI), Center for Global Health Research, Nairobi, Kenya; 5 Department of Paediatrics and Child Health/Kenyatta National Hospital, University of Nairobi, Nairobi, Kenya; 6 Institute of Tropical Medicine, Antwerp, Belgium

**Keywords:** *Bordetella pertussis*, *Chlamydia trachomatis*, cytomegalovirus, herpes virus type 1 and 2, Kenya, parvovirus B19, rubella, TORCH, *Toxoplasmosis gondii*, *Treponema pallidum*, varicella zoster virus

## Abstract

Women infected during pregnancy with TORCH (Toxoplasmosis, Other, Rubella, Cytomegalovirus, and Herpes simplex viruses) pathogens have a higher risk of adverse birth outcomes including stillbirth / miscarriage because of mother-to-child transmission. To investigate these risks in pregnant women in Kenya, we analyzed serum specimens from a pregnancy cohort study at three healthcare facilities. A sample of 481 participants was selected for TORCH pathogen antibody testing to determine seroprevalence. A random selection of 285 from the 481 participants was selected to measure seroconversion. These sera were tested using an IgG enzyme-linked immunosorbent assay against 10 TORCH pathogens. We found that the seroprevalence of all but three of the 10 TORCH pathogens at enrollment was >30%, except for *Bordetella pertussis* (3.8%), *Treponema pallidum* (11.4%), and varicella zoster virus (0.5%). Conversely, very few participants seroconverted during their pregnancy and were herpes simplex virus type 2 (*n* = 24, 11.2%), parvovirus B19 (*n* = 14, 6.2%), and rubella (*n* = 12, 5.1%). For birth outcomes, 88% of the participant had live births and 12% had stillbirths or miscarriage. Cytomegalovirus positivity at enrolment had a statistically significant positive association with a live birth outcome (*p* = 0.0394). Of the 10 TORCH pathogens tested, none had an association with adverse pregnancy outcome.

## Introduction

Pathogens that cause congenital infections worldwide are referred to as TORCH pathogens. Initially, TORCH pathogens have primarily included *Toxoplasmosis gondii*, other (syphilis, varicella zoster virus, parvovirus B19) *r*ubella, *c*ytomegalovirus (CMV), and *h*erpes simplex virus type 1 and 2 (HSV-1 and HSV-2) when detected during the early stages of pregnancy. Over the years, this list of pathogens has grown significantly to include, most recently, Zika virus (ZIKV), *Chlamydia trachomatis*, and human immunodeficiency virus (HIV). Depending on when the expecting mother is exposed to any of these pathogens, a myriad of different adverse outcomes may arise. Some of the notable adverse outcomes upon exposure during the first trimester of pregnancy include congenital malformations, intrauterine growth restriction or foetal death, whereas exposure during late pregnancy (i.e., third trimester) may result in adverse outcomes that present later after delivery.

In this study, we aimed to detect pre-existing antibodies against TORCH pathogens in pregnant women to understand previous exposure to these pathogens, susceptibility, and risk factors associated with infection of any of these pathogens during pregnancy. This information could tailor control and prevention programmes by public health authorities towards these specific high-risk pathogens in Kenya. However, some of these TORCH pathogens can reinfect the host or reactivate in regions where they lie dormant within the host, such as several of the *Herpesviridae* family of viruses (i.e., HSV-1, HSV-2, CMV, and VZV). In sub-Sahara African countries such as Kenya, there is limited information on the burden and epidemiology of these pathogens in pregnant women. A recent study in the Kenya coastal region identified a 1.9% microcephaly birth outcome rate that was not associated with the ZIKV in newborns [1]. For these microcephaly cases, the causative aetiology was not determined; however, the authors state that most of these microcephaly cases were also in infants determined to be small for gestational age (SGA). Of the limited information available from many African countries, epidemiological studies have shown that the burden of disease is greatest, where TORCH infections significantly contribute to prenatal and infant morbidity and mortality.

We conducted a study on ZIKV among pregnant women in Mombasa, Kenya. This ZIKV pregnancy cohort study was designed to detect active transmission of ZIKV and determine if infection during pregnancy with the African strain of ZIKV caused adverse birth outcomes [2]. Using this pregnancy cohort study, we conducted a secondary analysis to determine the presence of other TORCH pathogens (excluding ZIKV) and their association with adverse birth outcomes. Thus, we determined the seroprevalence of 10 different TORCH pathogens within this pregnancy cohort and their association with adverse birth outcomes. In addition, we measured the seroconversion rate for these pathogens during the participant’s pregnancy and matched this information to observed adverse birth outcomes. A secondary objective was to examine the seroprevalence by maternal age and HIV status as predictors of infection with TORCH pathogens.

## Methods

### Pregnancy cohort

**Figure 1.**
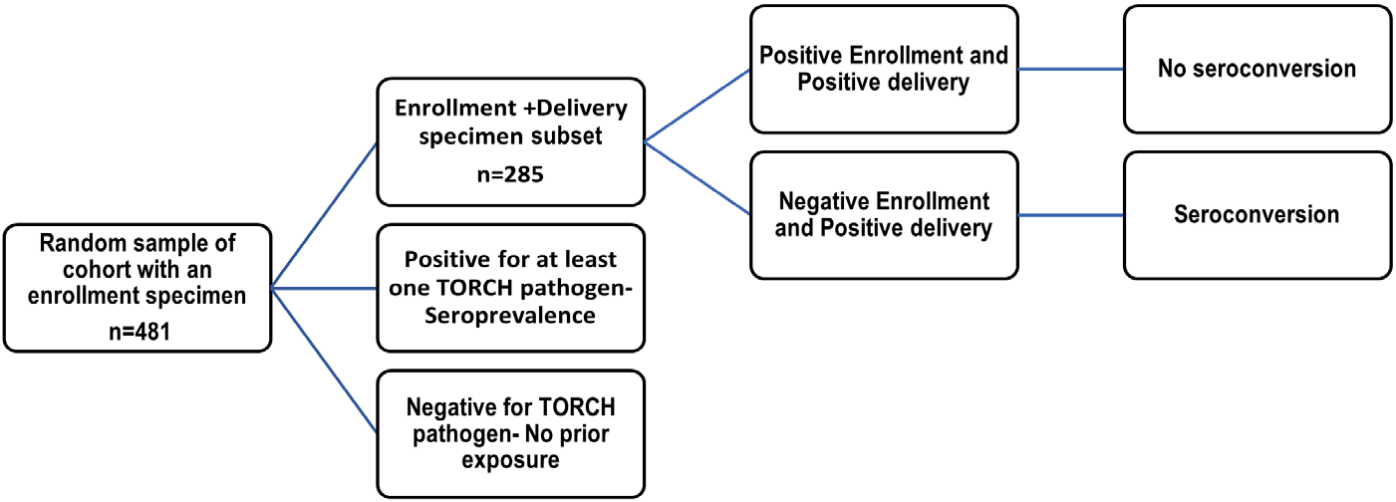
Participant selection criteria, testing, and outcome schematic.

From October 2017 to March 2019, we consented and enrolled pregnant women at <28 weeks estimated gestational age visit and ≥ 15 years of age that presented at antenatal clinics in 3 hospitals: Bomu, Coast General, and Port Ritz hospital (1 private and 2 public, respectively) in Mombasa, Kenya. Each enrolled participant was administered a questionnaire and was evaluated clinically, an obstetric ultrasound was conducted, and participant was asked to provide a blood sample of 5 mL at enrolment and then monthly throughout their pregnancy until delivery. During the first antenatal care (ANC) visit, we collected a urine specimen as well as clinical, obstetric history, demographic, socio-economic status, and exposure to mosquito bites from a total of 2,312 participants. Monthly follow-up visits were performed where the participants completed a questionnaire on risk factors and submitted a 5 ml blood specimen. During these follow-up visits, participants were asked of any symptoms of fever or rash within the last 7 days as part of the ZIKV monitoring. After delivery, we collected birth outcome data, including stillbirth and miscarriage, and postnatal parameters (such as head circumference, length, and birth weight) within 24 h of birth. We did not measure neurological outcomes such as hearing loss often associated with CMV infections. We randomly selected 481 women from the pregnancy cohort study who were ZIKV-negative with an enrolment specimen (Figure 1). A subset of women of the initial random selection (*n* = 285) were selected based on the availability of both enrolment and delivery specimens. From this subset, whose enrolment specimen for each TORCH pathogen was seronegative, were selected to assess for seroconversion of TORCH pathogens.

### Laboratory diagnostic testing

Commercial enzyme-linked immunosorbent assays (ELISAs) were used to detect IgG antibodies against 10 different TORCH pathogens (Euroimmun, Germany). The 10 TORCH pathogens included: *T. gondii*, cytomegalovirus (CMV), *Bordetella pertussis* (*B. pertussis*), parvovirus B19, herpes simplex viruses 1 and 2 (HSV-1, 2), rubella, *Treponema pallidum*, varicella zoster virus (VZV), and *C. trachomatis.* Both enrollment and delivery blood specimens from the participants were tested for the presence of IgG for all 10 TORCH pathogens. Sera collected from 5 mL of venous blood were separated by centrifugation, and 5ul of serum from each participant was tested by ELISA in duplicates according to the manufacturer’s recommendations. See Supplementary Appendix for manufacturer’s validation studies for each TORCH test.

### Statistical analysis

The study cohort enrolled 2,312 participants, among whom 1967 (85.1%) formed the sampling frame for the current study because they had complete outcome data (birth weight, miscarriage, stillbirth, live birth). Previous studies have reported TORCH seroprevalence estimates ranging from 2 to 98% in Kenya [3, 4]. Using this information, we assumed a seroprevalence of at least 6% for the TORCH pathogens tested, an absolute precision level of 2%, and applied finite population correction for a minimum sample size of 425.

We determined seroprevalence, defined as the proportion of participants who tested positive for any of the TORCH pathogens at enrollment. Furthermore, seroconversion was defined as a participant who tested negative for any of the 10 TORCH pathogens at enrollment, followed by a positive test at delivery. We described the study variables (demographic variables and pathogen status) using frequencies and percentages (95% confidence interval (CI)), and graphically using bar charts. Seroprevalence and seroconversion were calculated by maternal age category, HIV status, and SGA status. SGA was defined as a birth weight *z*-score of ≤ 1.28 at birth (equivalent to the 10th percentile) and extreme SGA as a birth weight *z*-score of ≤ 1.88 (equivalent to the 3rd percentile) based on INTERGROWTH-21st standards, and the corresponding *p*-values were estimated using either Pearson chi-square or Fisher’s exact test [5]. For participants who had birth outcome data, we investigated the association of each pathogen with adverse birth outcomes (stillbirth or miscarriage) using logistic regression and quantified the association with the odds ratio (OR). We calculated the 95% CI for the OR estimate. A multivariable regression analysis was not performed on this dataset because no independent variables reached a *p* < 0.1 cut-off value. The analyses were conducted using SAS software version 9.4 (SAS Institute Inc., Cary, NC). The level of significance was defined as *α* ≤ 0.05.

## Results

### The study

A total of 481 participants who were ZIKV-negative and had an enrollment specimen were selected using simple random selection from the pregnancy cohort and tested for 10 TORCH pathogens. A second subset of 285 participants who had both an enrollment specimen and a delivery specimen available were tested for the 10 TORCH pathogens to determine seroconversion rates. ZIKV-positive specimens were excluded from this study analysis and previously published [2]. A total of 62 (13.4%) of the women were ≤ 20 years old, 333 (71.9%) were between the ages of 21–35 years old, and 68 (14.7%) were > 35 years old. There were 130 (27%) enrolled during their first trimester, 296 (61.5%) that were enrolled within the second trimester (14–26 weeks), and 55 (11.4%) enrolled during their third trimester (Table 1).

**Table 1.** Participants demographic characteristics from single specimen taken at enrollment (*N* = 481) and participants with two-specimens obtained at enrollment and delivery (*N* = 285)

	Enrollment (*N* = 481)		With enrollment and delivery (*N* = 285)	
Demographic characteristics	*n*	Percent (95% CI)	*n*	Percent (95% CI)
Education				
Primary and below	119	27.5 (23.3; 31.7)	79	28.3 (23.1; 34.0)
Secondary	187	43.2 (38.5; 48.0)	118	42.3 (36.4; 48.3)
Tertiary	127	29.3 (25.1; 33.9)	82	29.4 (24.1; 35.1)
Occupation				
Employed	188	42.6 (38.0; 47.4)	118	41.4 (35.6; 47.4)
Self-employed	70	15.9 (12.6; 19.6)	52	18.3 (13.9; 23.2)
Unemployed	183	41.5 (36.9; 46.3)	115	40.4 (34.6; 46.3)
Site				
Bomu	150	34.0 (29.6; 38.6)	97	34.0 (28.6; 39.9)
Coast General	132	29.9 (25.7; 34.4)	90	31.6 (26.2; 37.3)
Port Reitz	159	36.1 (31.6; 40.7)	98	34.4 (28.9; 40.2)
HIV status				
Yes	85	20.7 (16.9; 25.0)	62	23.2 (18.3; 28.8)
No	325	79.3 (75.0; 83.1)	205	76.8 (71.2; 81.7)
Maternal age (years)				
≤ 20	62	12.9 (10.0; 16.2)	10	3.5 (1.7; 6.4)
21–35	351	73.0 (68.8; 76.9)	229	80.4 (75.3; 84.8)
> 35	68	14.1 (11.2; 17.6)	46	16.1(12.1; 20.9)
Gestational age at enrolment (weeks)				
0–13 (1st trimester)	130	27.0 (23.1; 31.2)	60	21.1 (16.5; 26.3)
14–26 (2nd trimester)	296	61.5 (57.0; 65.9)	192	67.4 (61.6; 72.8)
27+ (3rd trimester)	55	11.4 (8.7; 14.6)	33	11.6 (8.1; 15.9)

### Participants’ demographics at enrollment

Fewer than half the women enrolled in this study had a secondary-school level of education, and almost equal numbers of women had either a primary or below (27.5%) or tertiary education (29.3%) (Table 1). Similar proportion of pregnant women was employed (42.6%) and unemployed (41.5%), and 15.9% was self-employed. There was also a similar distribution of participants from the three study sites in Mombasa (Bomu 34%, Coast General 29.9% and Port Reitz 36.1%). The majority were HIV-negative (79.3%), between 21 and 35 years old (71.9%,) and enrolled within the 2nd trimester of their pregnancy (61.5%).

### Seroprevalence: past exposure to 10 TORCH pathogens

**Figure 2.**
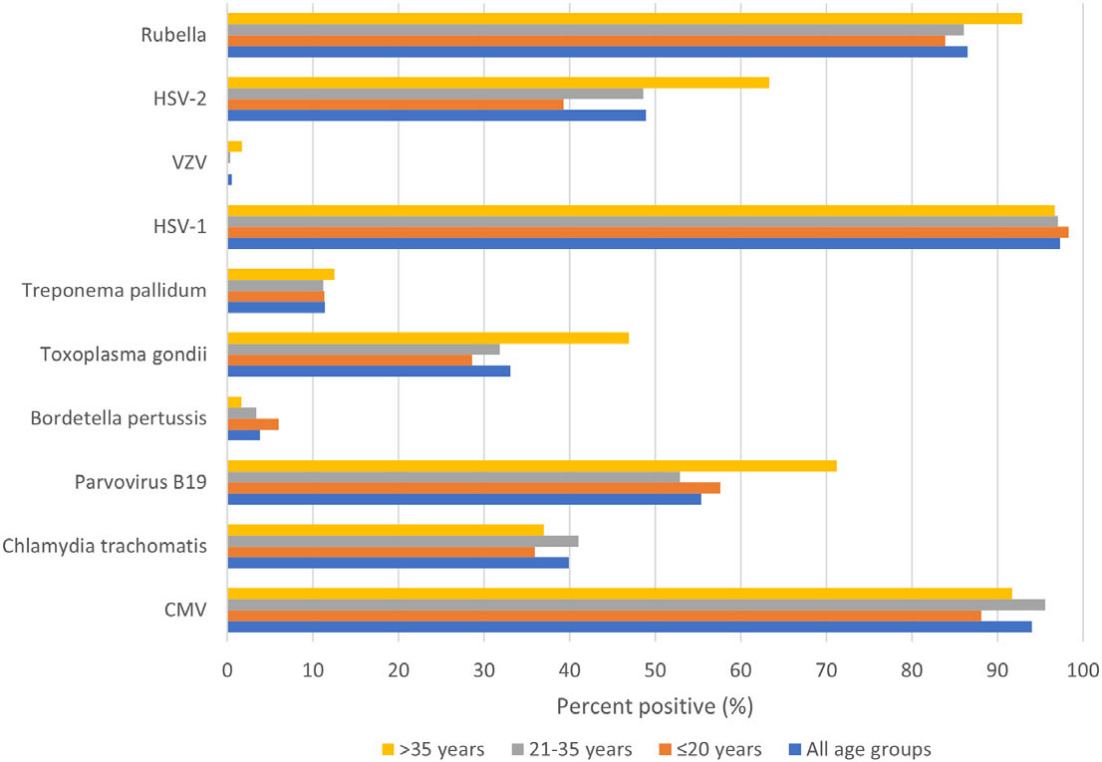
Seroprevalence of TORCH pathogens by age group. CMV, cytomegalovirus; HSV-1, herpes simplex virus type 1; HSV-2, herpes simplex virus type 2; VZV, varicella zoster virus.

Of the 481 pregnant women tested at enrollment, all had detectable IgG to at least one of the 10 TORCH pathogens tested (HSV-1 (97.3%), CMV (94%), rubella (86.5%), parvovirus B19 (55.4%), HSV-2 (48.9%), *C. trachomatis* (39.9%), *B. pertussis* (3.8%), *T. pallidum* (11.4%), and VZV (0.5%)) (Figure 2). All participants were simultaneously seropositive to at least two TORCH pathogens during either enrollment or delivery, driven primarily by HSV-1 and CMV (Table 2). Also, 78% of enrollment samples and 75% of delivery samples were positive for 4 to 6 TORCH pathogens.

**Table 2.** Seroprevalence of TORCH pathogens and the number of pathogens exposures from enrollment and delivery

TORCH target	Enrollment, *N* = 159^a^	Delivery, *N* = 159^a^
*Bordetella pertussis*	6 (3.8%)	4 (2.5%)
HSV-1	153 (96%)	157 (99%)
Chlamydia	61 (38%)	55 (35%)
CMV	155 (97%)	154 (97%)
HSV-2	78 (49%)	76 (48%)
Rubella	124 (78%)	130 (82%)
VZV	0 (0%)	0 (0%)
*T. gondii*	52 (33%)	50 (31%)
Parvo B19	80 (50%)	79 (50%)
*Treponema pallidum*	19 (12%)	14 (8.8%)
No. of TORCH pathogens that were seropositive		
2	8 (5.0%)	9 (5.7%)
3	18 (11%)	23 (14%)
4	50 (31%)	48 (30%)
5	49 (31%)	43 (27%)
6	26 (16%)	28 (18%)
7	7 (4.4%)	7 (4.4%)
8	1 (0.6%)	1 (0.6%)

CMV, cytomegalovirus; HSV-1, herpes simplex virus type 1; HSV-2, herpes simplex virus type 2; VZV, varicella zoster virus.

a *n* (%).

When categorizing the cohort by age, women ≤20 years old, representing the age group for adverse birth outcomes [6], had lower seroprevalence to CMV (88.1% vs. 94%), *C. trachomatis* (35.9% vs. 39.9%), *T. gondii* (28.6% vs. 33.1%), VZV (0% vs. 0.5%), HSV-2 (39.3% vs. 48.9%), and rubella (83.9% vs. 86.5%) compared to the overall rates for all age groups. The seroprevalence of those within the age group (21–35 years old) had similar or higher seroprevalence compared to the overall rates of the entire cohort for most of the 10 TORCH pathogens except for parvovirus B19 (52.9% vs. 55.4%), and *T. gondii* (31.8% vs. 33.1%). The age category (>35 years old) had higher seroprevalence than the overall rate of the entire cohort for parvovirus B19 (71.2% vs. 55.4%), *T. gondii* (46.9% vs. 33.1%), VZV (1.7% vs. 0.5%), HSV-2 (63.3% vs. 48.9%), and rubella (92.9% vs. 86.5%).

### Seroconversion to TORCH pathogens during pregnancy

**Figure 3.**
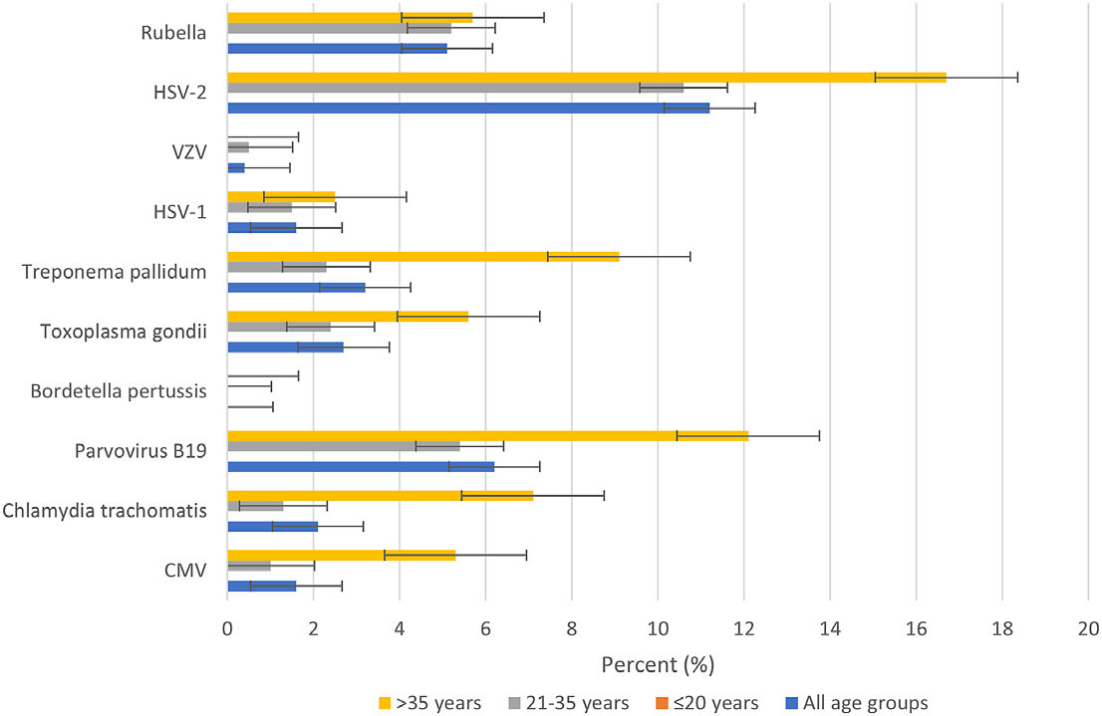
Seroconversion of TORCH pathogens by age group. Note that there were no seroconversions observed in age group <20 years old. CMV, cytomegalovirus; HSV-1, herpes simplex virus type 1; HSV-2, herpes simplex virus type 2; VZV, varicella zoster virus.

Of the 285 women that were tested at enrollment and delivery, we observed seroconversion to TORCH pathogens for HSV-2 (11.2%), parvovirus B19 (6.2%), rubella (5.1%), *T. pallidum* (3.2%), *C. trachomatis* (2.1%), CMV (1.6%), HSV-1(1.6%), and VZV (0.4%) (Figure 3). When we analyzed seroconversion results by age groups, we observed that none of the low-risk age groups of ≤20 years had seroconversion to any of the 10 TORCH pathogens. The medium-risk group (21–35 years old) had similar seroconversion results as the overall cohort, and the high-risk age group >35 years old had higher seroconversion for 7 of the 10 TORCH pathogens than the overall cohort: CMV (5.3% vs. 1.6%), *C. trachomatis* (7.1% vs. 2.1%), parvovirus B19 (12.1% vs. 6.2%), *T. gondii* (5.6% vs. 2.7%), *T. pallidum* (9.1% vs. 3.2%), HSV-1 (2.5% vs. 1.6%), and HSV-2 (16.7% vs. 11.2%).

### Birth outcomes related to seroprevalence of 10 TORCH pathogens

Of the 481 study participants selected for TORCH testing at enrollment, 362 (75.2%) had live births, 52 (10.8%) had miscarriages, 21 (4.4%) had stillbirths, and 46 (9.5%) did not have an outcome recorded (Table 3). CMV positivity at enrollment was the only serostatus group that had statistically significant decreased odds of non-live birth (OR = 0.378 (0.145; 0.985) and *p*-value = 0.0394).

**Table 3.** Distribution of pathogen status at enrollment by newborn outcomes non-live birth outcome status, the odds ratio quantifies the association of presence of an antibody to a pathogen at enrollment with the odds of a non-live birth outcome

Pathogen at enrollment		Outcomes		Odds of non-livebirth	
	Status	Non-livebirth (miscarriage/stillbirth)	Live birth	OR (95% CI)	*p*-value
Overall*		73	362		
CMV	Pos	62	305	0.378 (0.145; 0.985)	0.0394
	Neg	7	13		
*Chlamydia trachomatis*	Pos	26	119	0.954 (0.554; 1.642)	0.8648
	Neg	41	179		
Parvovirus B19	Pos	42	171	1.514 (0.876; 2.619)	0.1357
	Neg	24	148		
*Bortedella pertussis*	Pos	2	11	0.872 (0.189; 4.030)	0.8608
	Neg	64	307		
*Toxoplasma gondii*	Pos	12	66	0.857 (0.410; 1.793)	0.6821
	Neg	28	132		
*Treponema pallidum*	Pos	8	34	1.128 (0.496; 2.562)	0.7738
	Neg	58	278		
HSV-1	Pos	70	316	2.436 (0.309; 19.179)	0.3824
	Neg	1	11		
VZV	Pos	0	2	–	0.5240
	Neg	64	315		
HSV-2	Pos	37	145	1.373 (0.803; 2.346)	0.2458
	Neg	29	156		
Rubella	Pos	58	270	1.350 (0.579; 3.147)	0.4855
	Neg	7	44		

CMV, cytomegalovirus; HSV-1, herpes simplex virus type 1; HSV-2, herpes simplex virus type 2; VZV, varicella zoster virus.

–, OR could not be calculated due to 0 positive cases.

* The outcome status may not add up to the overall values when stratified by pathogen due to differential missingness.

When analyzing the seroprevalence of TORCH pathogens in women with the birth outcome of SGA, we found that, among live births, 225 (62.1%) deliveries had normal birth weight, 18 (5.0%) had low birth weight (<25,00 g), and 119 (32.8%) did not have birth weight recorded (Table 4). For pathogen seroprevalence by SGA, none of the TORCH pathogens were associated with a significant difference in the birth outcome of SGA.

**Table 4.** Pathogen seroprevalence for birth outcome of small for gestational age (SGA) among live births (*n* = 362)

Pathogen	SGA		*p*-value^a^
	No	Yes	
CMV	182/189 (96.3)	22/22 (100)	1.000
*Chlamydia trachomatis*	70/173 (40.5)	8/24 (33.3)	0.503
Parvovirus B19	99/188 (52.7)	12/23 (52.2)	0.965
*Bordetella pertussis*	5/195 (2.6)	1/23 (4.4)	0.492
*Toxoplasma gondii*	35/112 (31.3)	4/11 (36.4)	0.742
*Treponema pallidum*	21/186 (11.3)	4/21 (19.1)	0.294
HSV-1	191/195 (98.0)	24/24 (100)	1.000
VZV	0/188 (0.0)	0/25 (0.0)	1.000
HSV-2	88/180 (48.9)	7/21 (33.3)	0.177
Rubella	155/186 (83.3)	21/24 (87.5)	0.773

a *p*-value calculated using either Pearson chi-square or Fisher’s exact test.

### The association of HIV status and seroconversion and seroprevalence of 10 TORCH pathogens

We also determined the seroprevalence by HIV status and found that none of the *B. pertussis*- and VZV-positive cases were HIV-positive (Table 5). However, *T. gondii* (45.7% *p* = 0.046) and HSV-2 (73.2% *p* < .001) were significantly higher in HIV-positive women, whereas *B. pertussis* (0%, *p* = 0.046) was significantly higher in HIV-negative women. When comparing HIV status and seroconversion, only *T. gondii* was significantly higher among HIV-positive women (*p* = 0.03) (Table 6). Seroconversion for all other pathogens did not differ by HIV status.

**Table 5.** Pathogen seroprevalence by human immunodeficiency virus (HIV) status

Pathogen	HIV status		*p*-value^a^
	Yes	No	
CMV	73/75 (97.3)	273/290 (94.1)	0.386
*Chlamydia trachomatis*	23/72 (31.9)	109/269 (40.5)	0.185
Parvovirus B19	40/73 (54.8)	164/290 (56.6)	0.787
*Bordetella pertussis*	0/80 (0.0)	14/284 (4.9)	**0.046**
*Toxoplasma gondii*	21/46 (45.7)	52/180 (28.9)	**0.030**
*Treponema pallidum*	9/75 (12.0)	32/281 (11.4)	0.883
HSV-1	73/75 (97.3)	293/300 (97.7)	1.000
VZV	0/74 (0.0)	2/285 (0.7)	1.000
HSV-2	52/71 (73.2)	122/276 (44.2)	**<0.001**
Rubella	66/74 (89.2)	239/282 (84.8)	0.332

a *p*-value calculated using either Pearson chi-square or Fisher’s exact test.

Bold values are those with level of significance as defined as ɑ ≤ 0.05.

**Table 6.** Pathogen seroconversion by human immunodeficiency virus (HIV) status

Pathogen	HIV status		*p*-value^a^
	Yes	No	
CMV	0/52 (0.0)	3 /176 (1.7)	1.000
*Chlamydia trachomatis*	1/41 (2.4)	3/137 (2.2)	1.000
Parvovirus B19	5/44 (11.4)	8/165 (4.9)	0.153
*Bordetella pertussis*	0/53 (0.0)	0/163 (0.0)	–^b^
*Toxoplasma gondii*	3/30 (10.0)	1/111 (0.9)	**0.030**
*Treponema pallidum*	0/50 (0.0)	6/153 (3.9)	0.340
HSV-1	1/51 (2.0)	2/180 (1.1)	0.529
VZV	0/48 (0.0)	1/167 (0.6)	1.000
HSV-2	6/43 (14.0)	18/160 (11.3)	0.601
Rubella	5/52 (9.6)	7/169 (4.1)	0.159

a *p*-value calculated using either Pearson chi-square or Fisher’s exact test.

b *p*-value cannot be calculated due to zero counts.

Bold values are those with level of significance as defined as ɑ ≤ 0.05.

## Discussion

Our objective was to study the seroprevalence of TORCH pathogens using specimens collected from a cohort of pregnant women enrolled in a ZIKV study in Mombasa, Kenya. Our study showed detectable IgG antibodies in all women for at least one of the 10 TORCH pathogens. We found that there was relatively low seroconversion in this cohort for most of the TORCH pathogens, suggesting that few acquired an infection during their pregnancy; thus, risk factors associated with acute infections could not be calculated. Among those that had detectable seroconversion, the majority were women that fell within the two ages categories (21–25 and > 35 years old) compared to the ≤20 years old. Overall, *B. pertussis* had very low seroprevalence (3.8%) and no seroconversion, whereas HSV-1 (97.3%), CMV (94%), and rubella (86.5%) had the highest seroprevalence, and HSV-2 (11.2%), parvovirus B19 (6.2%), and rubella (5.1%) had the highest seroconversion within this cohort. Neither CMV and rubella seroprevalence were associated with SGA or HIV status; however, CMV was significantly associated with live birth outcome, suggesting that prior exposure was protective.

There were two viruses, of the four tested in this study, belonging to the *Herpesviridae* family (HSV-1 and CMV) had the highest percentage of seropositives within this cohort. This family of viruses are well adapted to their human host and thus highly infectious even within the general population. HSV-1 antibodies were detected in 97.3% of all the women selected for testing and had the highest seroprevalence of all the pathogens tested. Although HSV-1 had the highest seroprevalence within this cohort, it had a very low seroconversion rate of 1.6%, perhaps suggesting that few of the study participants acquired HSV-1 during their pregnancy, and almost all had prior immunity upon entering the study. When comparing miscarriage or stillbirth with live birth outcomes for HSV-1, the majority of live births were seropositive for HSV-1 (96.6%) and within the category of stillbirths and miscarriages most were also seropositive for HSV-1 (98.6%) which may have falsely suggested that this adverse birth outcomes could have been associated with HSV-1. However, when we compared the live birth to non-live birth overall odds ratio, there was no significant difference between that observed with HSV-1. Interestingly HSV-2, another virus belonging to the *Herpesviridae* family, had a much lower seroprevalence (48.6%) compared to HSV-1 but had the highest seroconversion rate (11.2%) of all the TORCH pathogens tested and the majority of the detectable seroconversion were within the 21- to 35-year-old group (75%).

CMV from the *Herpesviridae* virus family had the second highest seroprevalence (94%) compared to the other TORCH pathogens tested in our cohort. CMV is a very common infection with an overall seroprevalence among adults in the United States of America of 50% [7] and has been found to be even higher in LMIC [8]. The greatest risk to the foetus is found with exposure to CMV within the first trimester of pregnancy compared to the second or third trimesters [9]. Primary CMV infection can cause a range of clinical symptoms, including permanent hearing and vision loss, neurological impairments, and even death by miscarriage [10]. CMV is the leading cause of long-term disabilities in children and the leading cause of hearing loss worldwide. In pregnant women, the most common source of transmission of CMV is either sexual transmission or contact with urine or saliva from young children [11]. Previous studies in Africa indicated that there is an overall high prevalence of CMV in pregnant women, demonstrated in multiple African countries, including Kenya [12–15]. A study in Thika, Kenya, showed 77% prevalence with 8% IgM positivity suggesting recent infection [14]. Although the prevalence of CMV in our study was much higher (94%) than previously shown in Kenya, we only had 4 women (1.6%) who seroconverted in this cohort, suggesting that few of the expecting mothers were either infected early in their pregnancy prior to our sampling or perhaps had prior immunity to CMV. We suspect the latter since high seroprevalence was associated with lower odds of non-live birth (stillbirth or miscarriage) that was found to be significant (OR = 0.378 *p*-value 0.0394) compared to the overall birth outcomes within this cohort. Unfortunately, we were unable to measure infant outcomes such as hearing loss or other neurological impairments following delivery. The high seroprevalence of CMV may be associated with household size related to transmission within this setting [16].

Rubella had the third highest seroprevalence (86.5%) and the third highest seroconversion rate (5.1%) in our cohort. Rubella vaccination was incorporated into Kenya’s national childhood immunization schedule in 2016. Therefore, Rubella exposure or seroconversion among study participants was likely due to infection rather than vaccination. Women who have no immunity to rubella are at very high risk for congenital rubella infection and potentially adverse outcomes (congenital rubella syndrome (CRS)). CRS can only be detected many months after the baby is born; we were unable to determine if any of the infants in our cohort had CRS since we did not follow the women and infants after delivery. Although the exact number of CRS cases is unknown due to the lack of CRS surveillance, prior studies indicated that 6% of measles/rubella cases were women of reproductive age in Kenya [17]. Most participants were enrolled during the second trimester when the risk of CRS following rubella infection is low. The rubella vaccine programme for Kenya has had various interruptions since 2002. In 2016, Kenya introduced the combined measles and rubella (MR) vaccine in an attempt to improve vaccine coverage, but the coverage in pregnant women is not well documented, including in our study cohort participants [18]. Since our study was conducted between 2017 and 2019, the participants were not eligible to receive the rubella vaccine through the Kenya routine vaccination (https://www.afro.who.int/news/kenya-rolls-out-massive-measles-rubella-and-tetanus-campaign). The results from this study may provide sufficient information to estimate the seroprevalence of rubella and support policy for strengthening vaccination efforts in Kenya.

In our study, *T. pallidum* had a relatively high seroprevalence of 11.4% compared to that observed in North America (0.67%) [19]. A recent study by Warnecke et al. [20], demonstrated that the average seroprevalence of *T. pallidum* was between 1% and 3% in 6 different countries (Mexico, China, Poland, Germany, Brazil, and Turkey), demonstrating that this cohort in Kenya had between 3- and 10-fold higher levels in comparison with all 6 countries. Overall countries in Africa tend to have much higher prevalence of syphilis than the global average ranging from an average of 4.6% in Eastern African countries to 6.5% in Southern African countries [21]. Although our results are ~2-fold higher than the average reported prevalence for the East African region, the ELISA used in our study was validated by the manufacturer with 139 clinical specimens using the gold standard TPHA test, and the performance of their test indicated 100% sensitivity and specificity. One of the most surprising findings from this study was the very low seroprevalence (0.5%) and seroconversion (0.4%) of VZV among pregnant women in Kenya. Again, in Warnecke et al., the authors reported that all 6 countries tested had very high VZV seroprevalence with a mean of 95.9% and a range of 92.3–99.4% [20]. There are very few studies of prevalence of VZV in Kenya; however, a study by Hussey et al. [22] showed a VZV prevalence of 23% which is also significantly lower than detected globally [22, 23]. Our findings are significantly lower than even the Hussey et al., study and this could be due to the ELISA test parameters used to measure anti-VZV IgG antibodies. The manufacturer may have used a higher titre antibody cut-off to detect acute cases. Although the test application is to confirm a VZV suspected infection and/or reactivation, the manufacturer also claims that the test can determine immune status in early pregnancy. Nevertheless, our results are much lower than expected and must be taken with caution and requires further verification of this finding which we were unable to perform.

There were study limitations that should be considered. First, most of the cases were enrolled in the second trimester, thus missing the window of identifying potential seroconversions that may have happened earlier in the pregnancy. Also, we relied on seroconversion as a surrogate marker for acute infection leading to vertical transmission to the foetus. Second, most of the cohort fell into a single age category of 21–35 years old (71.9%), where age-related adverse outcomes are not commonly observed. Third, the vaccine status for some of the vaccine-preventable diseases was not known. Fourth, a full description of poor infant outcomes, such as hearing, or vision loss was outside of the scope of our study, thus limiting our analysis. Our analysis of co-infection used seroprevalence results due to the limited seroconversions observed; however, co-infections with any of these TORCH pathogens often culminate into a higher risk of adverse birth outcomes especially spontaneous abortions [24]. The causes of stillbirth and miscarriage could not be further determined, and only 66 stillbirths were examined at delivery with no gross anomalies noted. Finally, the results may need to be interpreted with caution since multivariable models could not be used because no independent variables reached a *p* < 0.1 cut-off value.

In summary, this is the first study to measure 10 TORCH pathogens in a large pregnancy cohort in Kenya. The seroprevalence of many of the TORCH pathogens in this cohort resembled those findings in Brazil except for *T. pallidum* and VZV. We found that only CMV antibodies at enrolment was negatively associated with adverse birth outcomes; however, prior exposure to other TORCH pathogens was not associated with adverse pregnancy outcomes. These findings support the inclusion of routine screening for those TORCH pathogens in national guidelines, especially those with high seroprevalence, where there are vaccines or prophylactic treatments available.

## Supporting information

Hunsperger et al. supplementary material Hunsperger et al. supplementary material

## Data Availability

The datasets used and/or analyzed during the current study are available from the corresponding author on reasonable request.

## Supplementary material

For supplementary material accompanying this paper visit http://doi.org/10.1017/S0950268824000165. click here to view supplementary material
