# Supplementary material for: Seroconversion and seroprevalence of TORCH infections in a pregnant women cohort study, Mombasa, Kenya, 2017–2019
Source: Epidemiol Infect. 2024 Feb 2;152:e68. doi: 10.1017/S0950268824000165 (PMC11077605; doi:10.1017/S0950268824000165)
Supplement: Hunsperger et al. supplementary material [file S0950268824000165sup001.docx]

**Appendix -All ELISAs were manufactured by Euroimmun (Germany)**

**Bordetella pertussis:** ELISA detecting Anti-IgG Bordetella pertussis toxin pertussis toxin (PT) antigen. (Cat# EI 2050-9601G)

**Detection Limit:** 0.2 IU/ml

**Cross reactivity:** None detected

**Test characteristics:** The Anti-Bordetella pertussis toxin IgG is based on species specific pertussis toxin (PT). Anti-PT antibodies are produced specifically after infection and vaccination with B. Pertussis. The test is calibrated using the International WHO standard serum and is in accordance with the latest guidelines for the serological diagnosis of B. pertussis infections. Recent vaccination (<1 year) should be taken into consideration when interpreting the results.

**Parvovirus B19:** ELISA detecting Anti-Parvovirus B19 IgG from recombinant viral structural protein expressed in eukaryotic cells (Cat# EI 2580-9601 G)

**Detection limit:** 0.1 IU/ml

**Cross reactivity:** None detected

**Test characteristics:** This ELISA detects anti- Parvovirus B19 IgG against the virus structural protein. The controls for the Anti-Parvovirus B19 ELISA (IgG) were calibrated using WHO International Standards for anti-parvovirus B29 plasma.

**Chlamydia trachomatis:** ELISA detecting Anti-Chlamydia trachomatis IgG (Cat# EI 2191 9601 G)

**Detection Limit:** 0.6 RU/ml

**Cross reactivity:** None detected

**Test characteristics:** This ELISA is based on anti-Chlamydia trachomatis MOMP antigen (major outer membrane protein) which is a transmembrane protein and the major part of the outer membrane of the elementary bodies. It is produced through protein purification with BGM cells infected with Chlamydia trachomatis of serotype K.

**Cytomegalovirus (CMV):** ELISA detecting Anti-CMV IgG using inactivated cell lysate of MRC-5 cells infected with AD169 CMV viral strain (Cat# EI 2570-9610 G).

**Detection limit:** 0.4 RU/ml

**Cross Reactivity:** None detected

**Test characteristics:** This ELISA is antigen coated plates containing lysate from MRC-5 infected with AD169 strain of CMV and is an anti-CMV IgG ELISA. There is no international reference serum available for antibodies against CMV.

**Rubella virus:** ELISA detecting anti-rubella virus IgG (Cat# EI 2590 9601 G)

**Detection Limit:** 0.3 IU/ml

**Cross Reactivity:** None detected

**Test characteristics:** An ELISA test anti-Rubella virus IgG from inactivated cell lysates of Vero cells infected with HPV-77 strain of Rubella virus. The assay was calibrated using international reference preparation NIBSC RUBI-1-94

**Herpes-simplex virus type 2 (HSV-2):** ELISA detecting anti-HSV-2 glycoprotein G2 IgG (Cat# EI 2532-9601-2G)

**Detection Limit:** 1.4 RU/ml

**Cross Reactivity:** None detected

**Test characteristics:** HSV-2 glycoprotein G2 is the antigen used for this Anti-HSV-2 IgG ELISA and allows for type-specific detection of IgG antibodies against HSV-2. No international reference serum exists for antibodies against HSV-2 thus the test was calibrated using relative units (RU). The plates were coated with HSV-2 glycoprotein G2 which is an envelope protein and it was purified by affinity chromatography

**Varicella zoster virus (VZV):** ELISA detecting anti-VZV IgG **(Cat# EI 2650 9601 G)**

**Detection Limit:** 3 IU/ml

**Cross Reactivity:** None detected

**Test characteristics:** The antigen used was purified VZV protein from strain Ellen derived from infected human fibroblasts (NHDF). The test was calibrated using international reference serum W1044. This ELISA measures anti-VZV IgG used to determine immunity especially in determining the immune status in early pregnancy.

**Herpes-simplex 1 virus (HSV-1):** ELISA detecting anti-HSV-1 IgG (Cat#EI 2541-9601-2G)

**Detection Limit:** 1.1 RU/ml

**Cross Reactivity:** None detected

**Test characteristics:** This ELISA uses recombinant glycoprotein C1 (gC1) from HSV-1. This glycoprotein is a membrane protein is only present in HSV-1 but not HSV-2. There is no international reference serum that exists fir antibodies against HSV-1 thus the calibration was performed using relative units/ml of synthesized gC1 antigen.

**Treponema pallidum:** ELISA detecting anti-Treponema pallidum IgG (Cat# EI 2111-9601 G)

**Detection Limit:** 0.3 RU/ml

**Cross Reactivity:** None detected

**Test characteristics:** This ELISA uses a mixture of four different recombinant antigens Treponema pallidum antigens (p15, p17, p47 and TmpA) expressed in E.Coli to detect anti-Treponema IgG in human serum or plasma. There is no international reference serum available for antibodies against Treponema pallidum thus results are presented as relative units.

**Toxoplasma gondii:** ELISA detecting anti-Toxoplasma gondii IgG (Cat # EI 2410-9601 G)

**Detection Limit:** 0.3 RU/ml

**Cross Reactivity:** None detected

**Test characteristics:** This ELISA uses microplates coated with Toxoplasma gondii organism purified by density gradient centrifugation with detergent extraction. The calibration of the test used International Units using the 3^rd^ international preparation of the WHO for anti-Toxoplasma serum Human Code TOXM. Serological assays for Toxoplasma gondii are important for diagnosis since direct detection of Toxoplasma is rarely successful.
